# Editorial: Use of Artificial Intelligence to evaluate drug-related behavioral changes in rodents

**DOI:** 10.3389/fphar.2024.1396454

**Published:** 2024-04-19

**Authors:** Victor Fattori, Sara González-Rodríguez, Rafael González-Cano

**Affiliations:** ^1^ Vascular Biology Program, Department of Surgery, Boston Children’s Hospital-Harvard Medical School, Boston, MA, United States; ^2^ Pharmacology, Faculty of Medicine, The University Institute of Oncology of Asturias (IUOPA), University of Oviedo, Oviedo, Spain; ^3^ Department of Pharmacology, Faculty of Medicine and Biomedical Research Center, Neurosciences Institute, Biosanitary Research Institute ibs.GRANADA, University of Granada, Granada, Spain

**Keywords:** rodents, pharmacology, behavior, artificail intelligence (AI), drugs

The advent of Artificial Intelligence (AI) heralds a revolution scarcely imagined by researchers a mere decade ago. In the intricate dance of pharmacology, in which each new or repurposed compound pirouettes through a meticulous choreography of testing and trials, AI might help identify better fit/effective drug candidates for different diseases. As we stand on the precipices of this brave new world, particularly in the subtle and often enigmatic realm of behavioral changes in our quintessential subjects: rodents.

Behavioral pharmacology has long been akin to an art as much as a science, requiring the discerning eye of the expert to detect and interpret the nuanced shifts in animal behavior post-administration of investigational drugs. However, while highly reliable when done by trained investigators, this traditional reliance on human observation is fraught with limitations—subjectivity, fatigue, and the inevitable variability that comes with manual scoring systems (Mogil, 2009). In the mouse grimace scale, the accuracy of scoring produced by an experienced investigator is over 97% compared to 72% of inexperienced ones (Langford et al., 2010). This demonstrates that highly trained investigators produce scores of higher consistencies in the mouse grimace scale. In addition, inexperienced investigators are likely to produce higher scores of mouse images than those originally predicted (Hohlbaum et al., 2020; Whittaker et al., 2021). Enter AI, with its promise of precision, consistency, and the capability to discern patterns beyond human recognition. The later development of a deep neural network to automate the grimace scale which scored with a similar accuracy of a highly trained human investigator, demonstrates that automated scorings can provide a reliable way to determine pain and pain relief in mice (Tuttle et al., 2018). Furthermore, there is a growing trend in preclinical pain research to use measures of physical and emotional function in animals experiencing chronic pain, aligning rodent behavioral phenotyping more closely with human experiences (Gonzalez-Cano et al., 2020). This alignment is crucial for improving the translation of preclinical results into clinical applications, as it seeks to capture a more comprehensive and clinically relevant assessment of pain and its effects. AI offers a means to automate and refine this alignment, ensuring that subtle but significant behavioral changes that signal pain relief or exacerbation are accurately captured and quantified. This editorial highlights the transformative potential of AI in rendering preclinical drug screening both precise and perceptive.

Quantifying animal behaviors automatically and without bias is one of the aspects that would facilitate the preclinical evaluation of drugs. One of the major problems is that evaluating a single frame makes it very difficult to discern if a particular behavior is occurring; information from preceding and following frames is required. An initial approach was seen using motion compression through another neural network that is included in the convolutional network (Bohnslav et al., 2021). In this article (*Automated Scratching Detection System for Black Mouse Using Deep Learning*), Sakamoto et al. they circumvent the problem by feeding the convolutional network multiple frames. This article represents a critical advancement in the objective assessment of pruritus, as evidenced by the development of an automated scratching detection system tailored for black mice. The use of a convolutional recurrent neural network (CRNN) exemplifies the potential of deep learning to overcome challenges related to phenotype recognition across different rodent strains. This system enhances the precision of behavioral analysis in drug screening, particularly for conditions associated with itching, and can significantly speed up the process of identifying potential therapeutic compounds.

The study from (*Subtle Alterations of Vestibulomotor Functioning in Conductive Hearing Loss*) Manno et al. extends the application of AI in pharmacology to the realm of sensory loss and its secondary impacts on behavior. By employing machine learning algorithms to analyze auditory brainstem responses and integrating these with behavioral assays, the research provides insights into the vestibulomotor deficits that may arise as a consequence of conductive hearing loss. This multidimensional approach to behavioral analysis can inform the development of drugs that address not just the primary sensory deficit but also its broader neurological ramifications.

The introduction of Drug-induced Behavioral Signature Analysis (DBSA) stands as a pioneer in the field, blending behavioral phenotyping with the concept of drug repurposing. By systematically sifting through high-content behavioral data to identify drugs that can potentially reverse phenotypes associated with rare diseases, this approach (*Enrichment Analysis of Phenotypic Data for Drug Repurposing in Rare Diseases*) Ambesi-Impiombato et al. embodies the innovative spirit of this Research Topic. The proof-of-concept study involving a Huntington’s Disease model paves the way for AI to play a central role in drug discovery, especially in areas with high disease burden and limited treatment options.

The development of free and open-source software capable of integrating data from diverse sources marks a significant leap in behavioral pharmacology research. This tool (*Integrated Software for Multi-Dimensional Analysis of Motion Using Tracking, Electrophysiology, and Sensor Signals*) Annavini et al. facilitates the rigorous and comprehensive analysis of motion and behavior in a streamlined and user-friendly manner. By automating the processing and analysis of complex datasets, this software enables researchers to extract more nuanced insights from their studies, potentially leading to the discovery of novel drug targets and the better understanding of drug effects on motor function.

The necessity for experienced evaluators is particularly relevant in the assessment of responses to evoked stimuli, especially in the evaluation of mechanical allodynia where von Frey filaments are heralded as the gold standard (Gonzalez-Cano et al., 2018). Various approaches have emerged to evolve their measurement using different technologies such as frustrated internal total reflection technology (FITR) (Zhang et al., 2022) or video recording to assess with von Frey filaments (Jones et al., 2020; Bohic et al., 2023). This article (*Quantification of Stimulus-Evoked Tactile Allodynia in Free Moving Mice by the Chainmail Sensitivity Test*) Ozdemir et al. addresses the challenge of assessing evoked tactile allodynia, a common symptom in many pain syndromes. The Chainmail Sensitivity Test (CST) provides a standardized and automated method to measure mechanical hypersensitivity. This novel test not only improves the throughput of behavioral assessments in models of chronic pain but also reduces the variability introduced by human operators. The CST’s correlation with established pain measures further underscores its potential as a reliable tool in the pharmacological evaluation of analgesics.

As this Research Topic eloquently showcases, AI is not merely an auxiliary tool but a central pillar in the modern edifice of pharmacological research. The contributions herein are not just a reflection of current scientific progress but rather a beacon guiding future explorations at the confluence of AI and pharmacology. By enhancing our understanding of drug-related behavioral changes in rodents, AI paves the way for more informed, precise, and ethical drug discovery, heralding a new era where the chasm between bench and bedside narrows ever so slightly, yet significantly.
